# Description of Changes in Crystal Orientations by the Elements of Logarithm of a Rotation Matrix

**DOI:** 10.1155/2017/4893956

**Published:** 2017-01-11

**Authors:** Susumu Onaka, Kunio Hayashi

**Affiliations:** Department of Materials Science and Engineering, Tokyo Institute of Technology, 4259-J2-63 Nagatsuta, Yokohama 226-8502, Japan

## Abstract

The logarithm ln⁡**R** of rotation matrix **R** is a skew symmetric tensor consisting of three independent elements of real numbers. In addition to the Euler angles and the axis/angle pair, the elements of ln⁡**R** called the log angles are also the set of three parameters of **R**. In this paper, we will show that the concept of the log angles is also useful to discuss changes in crystal orientations. The changes in **R** as a function of the position are given by the changes in the log angles. As an example, orientation changes caused by arrays of dislocations in a plastically deformed Cu single crystal are discussed.

## 1. Introduction

Electron backscatter diffraction analysis with scanning electron microscopy (SEM/EBSD) is a powerful technique to analyze orientations of crystalline materials. Using this technique, we can measure variations of crystal orientations in materials with high accuracy. Then, there is a chance to assess defect structures in grains such as dislocation structures that can vary crystal orientations around the defects [1], but it is necessary to express the variations in crystal orientations reasonably.

We can describe certain crystal orientation using a rotation matrix **R**, which is the 3 × 3 orthogonal matrix having nine elements [2]. However since the number of independent elements of **R** is three, sets of three parameters instead of **R** are more convenient to understand the rotation. To discuss variations **R** in crystalline materials obtained by results of SEM/EBSD measurements, appropriate parameters should be selected to show the relationship between changes in crystal orientations and microstructures formed in the materials.

The logarithm ln⁡**R** of **R** has been considered to discuss the rotation mathematically in the framework of the group theory [3–5]. The logarithm ln⁡**R** is a skew symmetric tensor consisting of three independent elements of real numbers. In addition to the Euler angles and the axis/angle pair [2], the elements of ln⁡**R** are also the set of three parameters of **R** [6]. In this paper, we will show that elements of ln⁡**R** called the log angles [7, 8] are useful parameters to discuss changes in crystal orientations. As an example, orientation changes caused by arrays of dislocations in a plastically deformed Cu single crystal are discussed.

## 2. Changes in Crystal Orientation

### 2.1. Logarithm of Rotation Matrix

When the axis/angle pair of **R** is given by a unit vector **n** = (*h*, *k*, *l*) and a rotation angle Φ, using the set of log angles (*w*_1_, *w*_2_, *w*_3_) for **R**, ln⁡**R** is written as [4]

(1) $$\begin{array}{c} \ln R=\left(\begin{array}{ccc} 0 & -w_{3} & w_{2}\\ w_{3} & 0 & -w_{1}\\ -w_{2} & w_{1} & 0 \end{array}\right)=\left(\begin{array}{ccc} 0 & -l\Phi & k\Phi \\ l\Phi & 0 & -h\Phi \\ -k\Phi & h\Phi & 0 \end{array}\right). \end{array}$$

On the other hand, the relationship between **R** and ln⁡**R** is written as [5]

(2) $$\begin{array}{c} R={\underset{p\to \infty }{\lim}\left(E+\frac{\ln R}{p}\right)}^{p}, \end{array}$$

where **E** is the unit matrix. Hence when *N* is a sufficiently large positive integer, we have

(3) $$\begin{array}{c} R\approx {\left(\;\delta R\;\right)}^{N}, \end{array}$$

where

(4) $$\begin{array}{c} \delta R\;=\;E+\frac{\ln R}{N}=\left(\begin{array}{ccc} 1 & -\frac{w_{3}}{N} & \frac{w_{2}}{N}\\ \frac{w_{3}}{N} & 1 & -\frac{w_{1}}{N}\\ -\frac{w_{2}}{N} & \frac{w_{1}}{N} & 1 \end{array}\right). \end{array}$$

**Figure 1:**
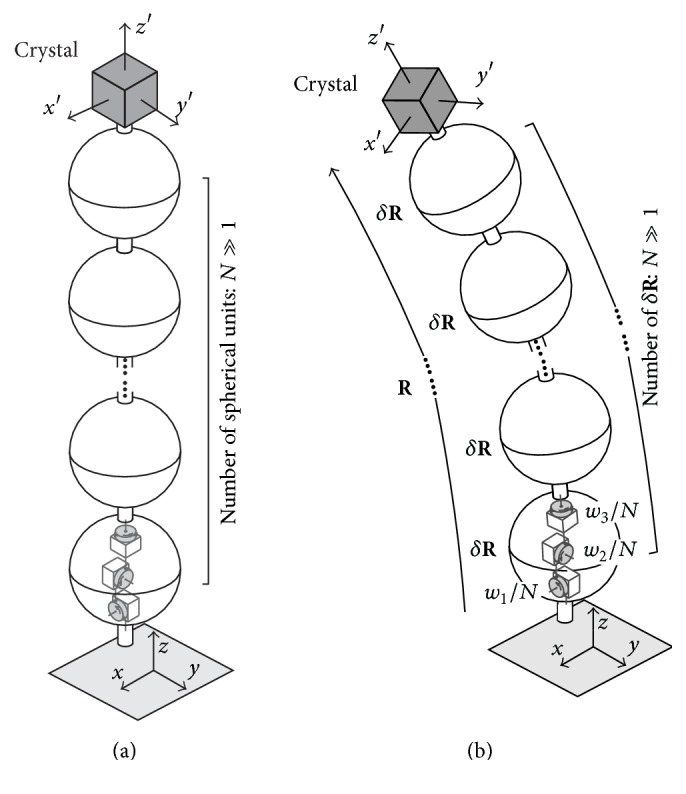
Graphical or mechanical representations of **R** given by (3). Spherical units corresponding to *δ ***R** given by (4) are stacked *N* times. (a) and (b) are the models for before and after the operation of **R**, respectively. In (b) **R** determines the primed coordinate axes of a crystal on the top of the model with respect to the reference unprimed frame.

Equation (4) shows that the *N*(≫1) times successive operations of *δ ***R** are equivalent to **R**. Hence, we have Figures 1(a) and 1(b) as graphical or mechanical representations [7, 8] of **R** given by (3) and (4). Spherical units corresponding to *δ ***R** with infinitesimal rotation angles are stacked *N* times. Since *δ ***R** is the rotation matrix with infinitesimal off-diagonal components, this can be shown as a product of three basic rotations in a spherical unit [7, 8]. These figures show that the log angles (*w*_1_, *w*_2_, *w*_3_) are the sums of the divided rotation angles around the coordinate axes of the reference frame and interpreted as the components of the rotation angles of **R** [7, 8]. This can be said for any **R** and is not limited to small angles.

Depending on selection of rotation axes and their orders, various sets of the Euler angles based on products of three basic rotations are defined for certain **R** [7]. The reason of the many sets is that the products of rotations are not commutative [7]. Different from the various sets of the Euler angles, a set of the log angles is uniquely determined for certain **R** [7]. Using a concept similar to that of the Euler angles, components of the rotation angles of **R** have been proposed by considering simple products of rotations [9]. However, as well as the Euler angles, the components given by considering the simple products of rotations may not be uniquely determined for certain **R**. The concept of the log angles can be applied to other characteristic values of rotations such as average orientations treated in previous studies [6, 10, 11] .

### 2.2. Log Angles for Small-Angle Rotation

**Figure 2:**
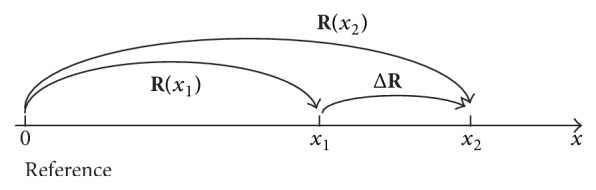
A model showing changes in crystal orientations for line scan of SEM/EBSD measurements.

As a model for line scan of SEM/EBSD measurements, here we consider changes in crystal orientations as shown by Figure 2. The crystal orientations at the positions *x*_1_ and *x*_2_ with respect to that at the origin O are given by **R**(*x*_1_) and **R**(*x*_2_), respectively. Here we define Δ**R** as the difference in the crystal orientations between those at *x*_1_ and *x*_2_ expressed by using the reference frame at the origin. Then the relation among the three rotation matrices is given by

(5) $$\begin{array}{c} R\left(\;x_{2}\;\right)=\Delta RR\left(\;x_{1}\;\right). \end{array}$$

**Figure 3:**
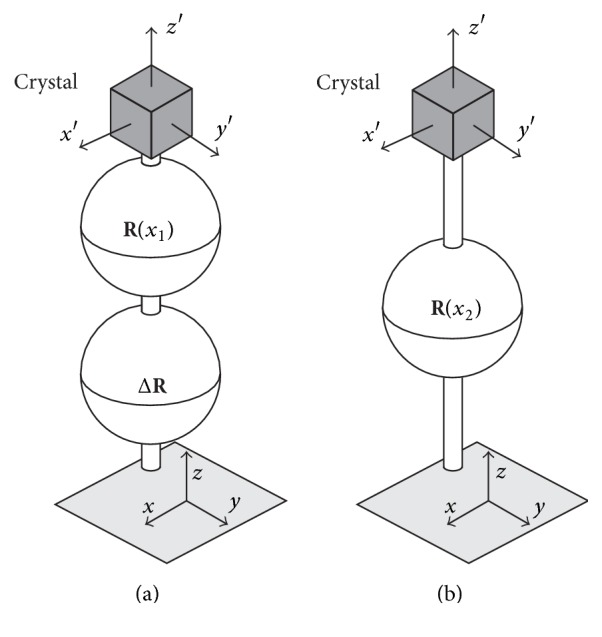
Graphical representations corresponding to the (a) right- and (b) left-hand sides of (5), where the difference Δ**R** in the crystal orientations between those at *x*_1_ and *x*_2_ is expressed by using the reference frame at the origin.

Figures 3(a) and 3(b) are graphical representations corresponding to the right- and left-hand sides of (5), respectively. Although Δ**R** is an additional rotation from **R**(*x*_1_) to **R**(*x*_2_), the stacking of the spherical units for the right-hand side of (5) becomes that as shown in Figure 3(a). If Δ**R** is the rotation defined by the rotated reference frame at *x*_1_, the order of the product in the right-hand side of (5) and the stacking of the spherical units in Figure 3(a) are changed.

Here we assume that Δ**R** is a small-angle rotation and the rotation angle ΔΦ of the axis/angle pair for Δ**R** satisfy |ΔΦ| ≪ 1. Then, (1) for Δ**R** is written from (2) to (4) as

(6) $$\begin{array}{c} \Delta R\approx E+\ln\Delta R. \end{array}$$

The log angles (Δ*w*_1_, Δ*w*_2_, Δ*w*_3_) of Δ**R** written as

(7) $$\begin{array}{c} \ln\Delta R=\left(\begin{array}{ccc} 0 & -\Delta w_{3} & \Delta w_{2}\\ \Delta w_{3} & 0 & -\Delta w_{1}\\ -\Delta w_{2} & \Delta w_{1} & 0 \end{array}\right) \end{array}$$

satisfy |Δ*w*_1_|, |Δ*w*_2_|, |Δ*w*_3_| ≪ 1 since |ΔΦ| ≪ 1. Using this equation, (5) is rewritten as

(8) $$\begin{array}{c} R\left(\;x_{2}\;\right)\approx \left(\begin{array}{ccc} 1 & -\Delta w_{3} & \Delta w_{2}\\ \Delta w_{3} & 1 & -\Delta w_{1}\\ -\Delta w_{2} & \Delta w_{1} & 1 \end{array}\right)R\left(\;x_{1}\;\right)\approx \left(\begin{array}{ccc} 1 & 0 & 0\\ 0 & 1 & -\Delta w_{1}\\ 0 & \Delta w_{1} & 1 \end{array}\right)\left(\begin{array}{ccc} 1 & 0 & \Delta w_{2}\\ 0 & 1 & 0\\ -\Delta w_{2} & 0 & 1 \end{array}\right)\left(\begin{array}{ccc} 1 & -\Delta w_{3} & 0\\ \Delta w_{3} & 1 & 0\\ 0 & 0 & 1 \end{array}\right)R\left(\;x_{1}\;\right). \end{array}$$

Equation (8) shows that the infinitesimal log angles (Δ*w*_1_, Δ*w*_2_, Δ*w*_3_) of Δ**R** can be interpreted as the components of rotation angles giving the difference in the crystal orientations between **R**(*x*_1_) at *x*_1_ and **R**(*x*_2_) at *x*_2_.

### 2.3. Position Dependence of Change in Crystal Orientation

Here we assume that the difference Δ*x* in *x*_1_ and *x*_2_ is small. When **R**(*x*) is differentiable with respect to *x*, the difference between **R**(*x*_1_) at *x*_1_ and **R**(*x*_2_) at *x*_2_ is written as

(9) Rx2−Rx1Rx+Δx−Rx≈dRxdxΔx.

From this equation and (5) to (7), we have

(10) $$\begin{array}{c} \left(\begin{array}{ccc} 0 & -\frac{\Delta w_{3}}{\Delta x} & \frac{\Delta w_{2}}{\Delta x}\\ \frac{\Delta w_{3}}{\Delta x} & 0 & -\frac{\Delta w_{1}}{\Delta x}\\ -\frac{\Delta w_{2}}{\Delta x} & \frac{\Delta w_{1}}{\Delta x} & 0 \end{array}\right)\approx \left[\;\frac{dR}{dx}\;\right]R^{-1}. \end{array}$$

As shown by this equation, the changes in the rotation matrix **R**(*x*) as a function of the position *x* are given by the changes in the log angles Δ*w*_1_, Δ*w*_2_, and Δ*w*_3_.

If all **R**(*x*) for any *x* are small-angle rotations, products of matrices relating to **R**(*x*) become commutative and ln⁡Δ**R** is written from (5) as

(11) $$\begin{array}{c} \ln\Delta R=ln\left[\;R{\left(\;x_{1}\;\right)}^{-1}R\left(\;x_{2}\;\right)\;\right]\approx \ln R\left(\;x_{2}\;\right)-\ln R\left(\;x_{1}\;\right). \end{array}$$

Then, a differential form for the small-angle rotations is given by

(12) $$\begin{array}{c} \frac{d\ln R}{dx}\approx \left[\;\frac{dR}{dx}\;\right]R^{-1}\approx R^{-1}\left[\;\frac{dR}{dx}\;\right]. \end{array}$$

## 3. Changes in Crystal Orientations of Cold-Rolled Cu Single Crystal

### 3.1. Cold Rolling of Cu Single Crystal and Crystal Orientations after Cold Rolling

**Figure 4:**
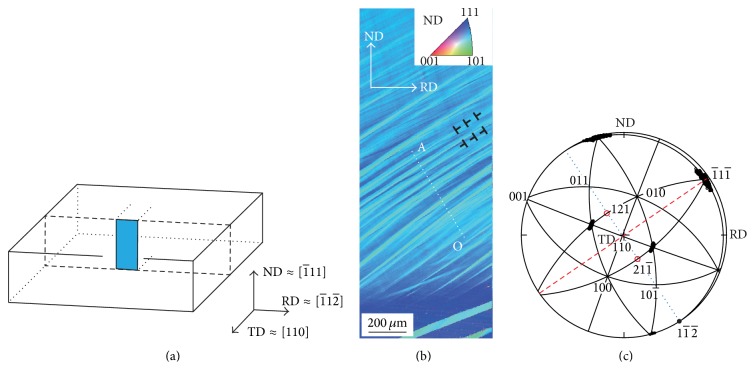
Cold rolling of Cu single crystal. (a) Schematic illustration showing a shape and crystallographic orientations before cold rolling. The region analyzed by SEM/EBSD technique after rolling is also shown in (a). (b) The inverse pole figure (IPF) map showing the crystallographic orientation of ND on the TD plane after rolling to 15% reduction in thickness. (c) The stereographic projection showing the orientation at the origin O after the rolling. Changes of crystal orientations along the line OA are also shown by {111} pole figures in (c).

As an example of applications of the present analysis, we consider changes in crystal orientations of a Cu single crystal caused by plastic deformation. A plate-like Cu single crystal (99.9% mass purity) with a 2 mm thickness and 20 mm width and length was cold rolled.  Figure 4(a) shows a schematic of the Cu single crystal before cold rolling. Directions TD, RD, and ND shown in this figure are the transverse, rolling, and normal directions of the applied rolling, respectively. TD, RD, and ND construct an orthogonal coordinate system and their crystallographic directions are also indicated in Figure 4(a).

We observed how the microstructure evolved after rolling to 15% reduction in thickness by SEM/EBSD technique. A JSM-7001F (JEOL) controlled by a program, OIM Data Collection ver. 7.01 (TSL), was used. The acceleration voltage and step size were 15 kV and 1.0 *μ*m, respectively. The cross-section normal to the TD (TD plane) was observed as shown by the rectangular region in Figure 4(a).  Figure 4(b) is the inverse pole figure (IPF) map showing the crystallographic orientation of ND on the TD plane of the rolled specimen. The color code for the IPF map is shown in a standard triangle at the top-right. The directions of RD and ND on the TD plane are also indicated.

As shown in Figure 4(b), almost parallel blue and green bands showing orientation splitting appeared after the rolling. Inclination of the bands is from 0.56 to 0.65 rad (32 to 37 deg.) to RD. We discuss orientation changes along the line from the origin O to A shown in this IPF map. Orientation measurements along the line were made at intervals of 2.4 *μ*m. The stereographic projection in Figure 4(c) shows the orientation at the origin O. Changes of crystal orientations along the line OA are shown by {111} pole figures in Figure 4(c). These show that the orientation changes caused by the rolling are mainly rotations around TD.

### 3.2. Changes in Log Angles

**Figure 5:**
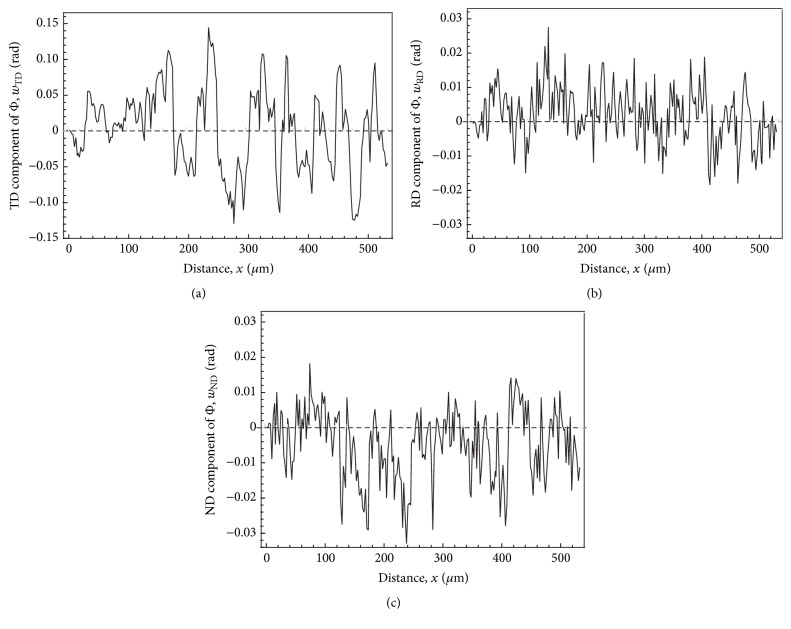
The variations of the log angles (*w*_TD_, *w*_RD_, *w*_ND_) along the line OA in Figure 4(b) as a function of the distance from the origin O. The log angles show the orientation changes from the orientation at the origin O. (a), (b), and (c) are for the components of *w*_TD_, *w*_RD_, and *w*_ND_, respectively.

Figure 5 shows the variations of the log angles (*w*_TD_, *w*_RD_, *w*_ND_) along the line OA as a function of the distance from the origin O. The log angles show the orientation changes from the orientation at the origin O. These show that the changes in *w*_TD_ are about ±0.14 rad and the largest among the three components. The periodic changes in *w*_TD_ show that the interfaces between the blue and green bands in Figure 4(b) are small-angle tilt boundaries causing clockwise and anticlockwise rotations around TD. Parallel directions of the traces of interfaces shown in Figure 4(b) are almost parallel to a red broken line from the center of the projection to $\overset{-}{1}1\overset{-}{1}$ in Figure 4(c). This red broken line is perpendicular to the dotted line showing directions perpendicular to $\left[\overset{-}{1}1\overset{-}{1}\right]$ in the same figure. These show that the small-angle tilt boundaries are composed of arrays of edge dislocations on parallel $\left(\overset{-}{1}1\overset{-}{1}\right)$ slip planes as indicated by dislocation marks at the middle-right of Figure 4(b). Considering the initial orientation of the Cu single crystal shown in Figure 4(a), we can say that this $\left(\overset{-}{1}1\overset{-}{1}\right)$ is the slip plane operated during the rolling [12].

**Figure 6:**
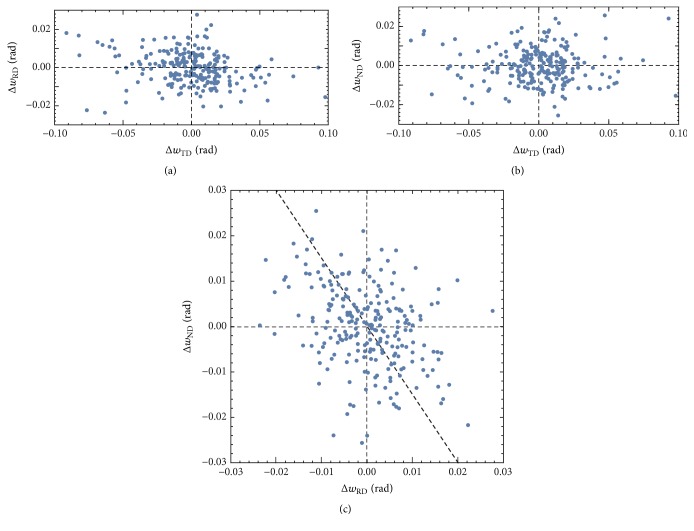
The relations among Δ*w*_TD_, Δ*w*_RD_, and Δ*w*_ND_ at the same locations on the line OA in Figure 4(b), where Δ*w*_TD_, Δ*w*_RD_, and Δ*w*_ND_ are the changes in the log angles between neighboring two measuring points. See text for an inclined broken line in (c).

Figures 6(a)–6(c) show the relations among Δ*w*_TD_, Δ*w*_RD_, and Δ*w*_ND_ at the same locations on the line OA in Figure 4(b), where Δ*w*_TD_, Δ*w*_RD_, and Δ*w*_ND_ are the changes in the log angles between neighboring two measuring points 2.4 *μ*m apart. In the Δ*w*_TD_ − Δ*w*_RD_ and Δ*w*_TD_ − Δ*w*_ND_ relations shown in Figures 6(a) and 6(b), the dispersion of data seems to be symmetric and random. However, in the Δ*w*_ND_ − Δ*w*_RD_ relation shown in Figure 6(c), the data disperses systematically near an inclined broken line in this figure. The slope of the inclined broken line is discussed later.

**Figure 7:**
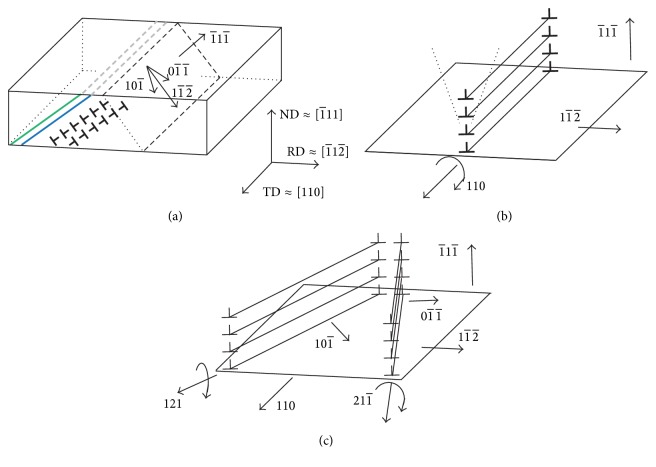
Schematic illustrations showing dislocation structures in the rolled single crystal. See text for the details.

Figure 7(a) schematically shows the small-angle tilt boundaries observed on the TD plane. Possible slip directions $\left[0\overset{-}{1}\;\overset{-}{1}\right]$ and $\left[10\overset{-}{1}\right]$ on $\left(\overset{-}{1}1\overset{-}{1}\right)$ are also indicated in Figure 7(a). During rolling, macroscopic shear direction on $\left(\overset{-}{1}1\overset{-}{1}\right)$ may be $\left[1\overset{-}{1}\;\overset{-}{2}\right]$ since this causes the plane-strain plastic deformation on the ND-RD plane. Such deformation is achieved by the double slip of $\left[0\overset{-}{1}\;\overset{-}{1}\right]$ and $\left[10\overset{-}{1}\right]$ on $\left(\overset{-}{1}1\overset{-}{1}\right)$. When shear deformation on $\left(\overset{-}{1}1\overset{-}{1}\right)$ to $\left[1\overset{-}{1}\;\overset{-}{2}\right]$ occurs, crystal rotations around TD [110] occur as shown in Figure 7(b). However, as shown in Figure 7(c), this deformation is the combination of the two shears $(\overset{-}{1}1\overset{-}{1})\;\left[0\overset{-}{1}\;\overset{-}{1}\right]$ and $\left(\overset{-}{1}1\overset{-}{1}\right)\;\left[10\overset{-}{1}\right]$ which cause crystal rotations around $\left[21\overset{-}{1}\right]$ and [121], respectively.

The axis (*h*, *k*, *l*) of rotation is related to the components of the log angles as shown by (1). The rotation between neighboring two measuring points along the line OA is caused by $\left[0\overset{-}{1}\;\overset{-}{1}\right]$ and $\left[10\overset{-}{1}\right]$ dislocations between them. The components of the log angles are proportional to the components of the rotation axis. The rotation axis may change between $\left[21\overset{-}{1}\right]$ and [121] on $\left(\overset{-}{1}1\overset{-}{1}\right)$ depending on the ratio of numbers of the $\left[0\overset{-}{1}\;\overset{-}{1}\right]$ and $\left[10\overset{-}{1}\right]$ dislocations. Hence, the change in the rotation axes is expressed by the Δ*w*_ND_ − Δ*w*_RD_ relation as shown by the broken line in Figure 6(c). We have considered the changes in crystal orientations in the rolled Cu single crystal with the changes in the log angles. The systematic dispersion of the Δ*w*_ND_ − Δ*w*_RD_ relation shown in Figure 6(c) is thus explained by the dislocation structures shown in Figure 7(c).

## 4. Conclusions

The logarithm ln⁡**R** of rotation matrix **R** is a skew symmetric tensor consisting of three independent elements of real numbers. The log angles are the elements of ln⁡**R** and the set of three parameters of **R**. We have shown that the concept of the log angles is useful to discuss changes in crystal orientations. The changes in crystal orientations as a function of the position are given by the changes in the log angles. As an example, orientation changes caused by arrays of dislocations in a cold-rolled Cu single crystal are discussed.
